# Innovative approaches for mycotoxin detection in various food categories

**DOI:** 10.1186/s13568-024-01662-y

**Published:** 2024-01-12

**Authors:** Marina H. Boshra, Ghadir S. El-Housseiny, Mohammed M. S. Farag, Khaled M. Aboshanab

**Affiliations:** 1 Department of Mycotoxins, Central Public Health Laboratories (CPHL), Ministry of Health, Cairo, Egypt; 2 Department of Microbiology and Immunology, Faculty of Pharmacy, Organization of African Unity St., Ain Shams University, Abbassia, PO: 11566, Cairo, Egypt; 3 Botany and Microbiology Department, Faculty of Science, Al-Azhar University, Cairo, 11884 Egypt; 4 Armed Forces College of Medicine (AFCM), Cairo, Egypt

**Keywords:** Mycotoxins, Chromatography, ELISA, And immunoaffinity, Biosensors, Biosensors, Fluorescent polarization

## Abstract

Mycotoxins (MTs), produced by filamentous fungi, represent a severe hazard to the health of humans and food safety, affecting the quality of various agricultural products. They can contaminate a wide range of foods, during any processing phase before or after harvest. Animals and humans who consume MTs-contaminated food or feed may experience acute or chronic poisoning, which may result in serious pathological consequences. Accordingly, developing rapid, easy, and accurate methods of MTs detection in food becomes highly urgent and critical as a quality control and to guarantee food safety and lower health hazards. In this review, we highlighted and discussed innovative approaches like biosensors, fluorescent polarization, capillary electrophoresis, infrared spectroscopy, and electronic noses for MT identification pointing out current challenges and future directions. The limitations**,** current challenges, and future directions of conventional detection methods versus innovative methods have also been highlighted and discussed.

**Supplementary Information:**

The online version contains supplementary material available at 10.1186/s13568-024-01662-y.

## Introduction

Since the discovery of the first MTs, aflatoxins (AFs), in 1965, there has been an upward trend in the publication of scholarly articles on MTs, with 16,821 papers being listed in Scopus. Data unmistakably demonstrated the importance of MTs research, nevertheless, in many low-income nations where MTs have an impact on staple foods, the MTs-related global health problem is still commonly disregarded (Wild & Gong 2009). Unfortunately, these locations represent the least controlled regarding farming methods and exposure to humans, resulting in long-term and frequently high amounts of exposure. Only the wealthier countries in the world have focused on adhering to strict import laws regarding MTs contamination (Battilani et al. 2016). The population in developing nations, particularly in rural regions, depends on locally produced foods and frequently faces issues with food security and MTs contamination, which is seen as a significant problem with food quality (Singh and Mehta 2020).

MTs are secondary metabolites of filamentous fungi, belonging to the *Ascomycota phylum*, with a low molecular mass (MW 700 Da) that endanger the health of both people and animals (Liew & Mohd-Redzwan 2018) (Alshannaq and Yu 2017). The incidence of the AF-caused Turkey X sickness, which claimed the lives of over 100,000 turkeys in 1960, sparked research in MTs. After that, it was discovered that Hepatocellular carcinoma (HCC) can develop because of AFs, which are carcinogenic in both people and animals (Liew & Mohd-Redzwan 2018). Since then, we discovered more than 400 distinct MTs with varied chemical compositions and characteristics that are produced by numerous different fungi species (Palumbo et al. 2020). *Penicillium, Alternaria, Claviceps, Aspergillus, Fusarium,* and *Stachybotrys* are the primary genera of mycotoxigenic fungus (Zain 2011). The most dangerous MTs are deoxynivalenol (DON), fumonisins (FBs), ergot alkaloids (EAs), T-2 and HT-2 toxins (T-2, HT-2) as well as aflatoxins (AFs), ochratoxin A (OTA), zearalenone (ZEN), enniatins (ENs), patulin (PAT), and *Alternaria* toxins (ATs) (Wokorach et al. 2021; Abrunhosa et al. 2001). MTs have been discovered to be present in a variety of agricultural goods, including wheat, barley, maize, oats, rice (Palumbo et al. 2020), vegetables, and fruits (Sanzani et al. 2016). Additionally, MTs can infect herbs (Sedova et al. 2018; Ałtyn et al. 2020), spices (Potortì et al. 2020), drinks such as wine, fruit juices, and beer (Quintela 2020), milk (Becker-Algeri et al. 2016), nuts (Kluczkovski 2019), coffee and cocoa (Bessaire et al. 2019; Huertas-Pérez et al. 2017). Various fungal species' development and MT generation processes can be influenced by a variety of variables. These include the surrounding environment, including its humidity, temperature, pH, water activity, substrate type, nutrients, physiological condition, level of inoculation, and microbial interactions (Brzonkalik et al. 2012; Agriopoulou et al. 2020). MTs production can take place during the preparation, packaging, distribution, and storage of agricultural products, or during the preparation of food (Karlovsky et al. 2016). Due to the environment, inadequate production methods, and poor storage conditions in developing nations, MTs contamination occurs more frequently in food and feed (Al-Jaal et al. 2019). Additionally, because many MTs are resistant to heat, chemical, and physical treatments, they are challenging to remove from food during processing (Marin et al. 2013). Numerous approaches have been put out to reduce the MTs contamination of various food products, but no definitive answers have been found.

MTs harm people’s and animals’ health, impede international trade, waste food and feed, and take money away from initiatives to address MTs’ problems through legislation, research, and enforcement (Stoev 2013). Unfortunately, every year, MTs infect over 25% of the world’s harvested crops, resulting in billion-dollar losses for business and agriculture (Marin et al. 2013). A recent study revealed that MTs are present in 60–80% of crops globally (Eskola et al. 2020). Both OTA and AFB1 were categorized by the International Agency for Research on Cancer (IARC) as being potentially carcinogenic to humans in Group 2B and Group 1, respectively while Trichothecenes and ZEN (Group 3) were not acknowledged as Human Carcinogens (Accessed on 12 November 2023). The World Health Organisation (WHO), the European Commission (EC) (https://eur-lex.europa.eu/legal-content/EN/TXT/PDF/?uri=CELEX:02006R1881-20140701&from=EN) (Accessed on 12 November 2023), the Food and Agriculture Organisation of the United Nations (FAO), and other national and international institutions and organizations have identified potential health risks to humans and animals associated with food- or feedborne MTs intoxication. They have addressed this issue by developing regulatory limits for major MTs classes and selected individual MTs types (Krska et al. 2008). Based on the health consequences of MTs, there is an urgent need for rapid, easy, and accurate methods of MTs detection in food as a quality control and to ensure food safety and lower health dangers. Accordingly, we highlighted and discussed the up-to-date innovative approaches that have been employed for MT detection pointing out current challenges and future directions. The limitations**,** current challenges, and future directions of conventional detection methods versus innovative methods have also been highlighted and discussed.

### Occurrence of mycotoxicosis

When exposure to mold toxins/substances results in poisoning, this condition is known as Mycotoxicosis. Mycotoxicosis can affect the health of people and animals in a variety of ways, including ingestion, inhalation, skin contact, lymphatic system entry, and bloodstream entry. While chronic impacts can take months, years, or even decades to appear, acute effects show up within 72 h of exposure. The type of MT determines the symptoms and effects of mycotoxicosis, although two or more MTs may have comparable effects (Bulgaru et al. 2021). When MTs are present in toxic doses, they typically have the following impacts on humans and animals: recognizable diseases, weakened immunity, mortality, and acting as irritants or allergens. Numerous MTs are toxic to other living things, including fungi and bacteria (Keller et al. 2005). The uncommon phenotypical sex changes in chickens, whereby they appear and behave as though they are male, have been attributed to MTs in stored animal feed (Melina 2020). By means of inhalation and absorption into the blood and lymphatic pathways, MTs infect humans (Bennett and Klich 2003). Mycotoxicosis symptoms depend on mycotoxin type, sex, age, and general health of the victims, as well as the amount of MT present and the duration of exposure (Claeys et al. 2020). Insufficient research has been done on the interactions between several elements, including food, genetics, and relationships with various toxins. As a result, there is a chance that mycotoxicosis will be made worse by vitamin deficiencies, alcoholism, calorie restriction, and viral infections (Bennett and Klich 2003). In the 1990s, MTs contributed to public health worries over the increasing number of mold settlements, which might have cost millions of dollars. This was a direct outcome of research conducted in Cleveland, Ohio, which gave proof of the association between MTs in infants’ pulmonary hemorrhage and the spores of *Stachybotrys* (Agriopoulou et al. 2020). The maximal concentration of MTs in research on dietary (nutritional) supplements derived from plants in 2015 was estimated to be around 37 mg per kg for the supplement based on milk thistle (Veprikova et al. 2015).

### Types of mycotoxins

#### Aflatoxins (AFs)

**Fig. 1:**
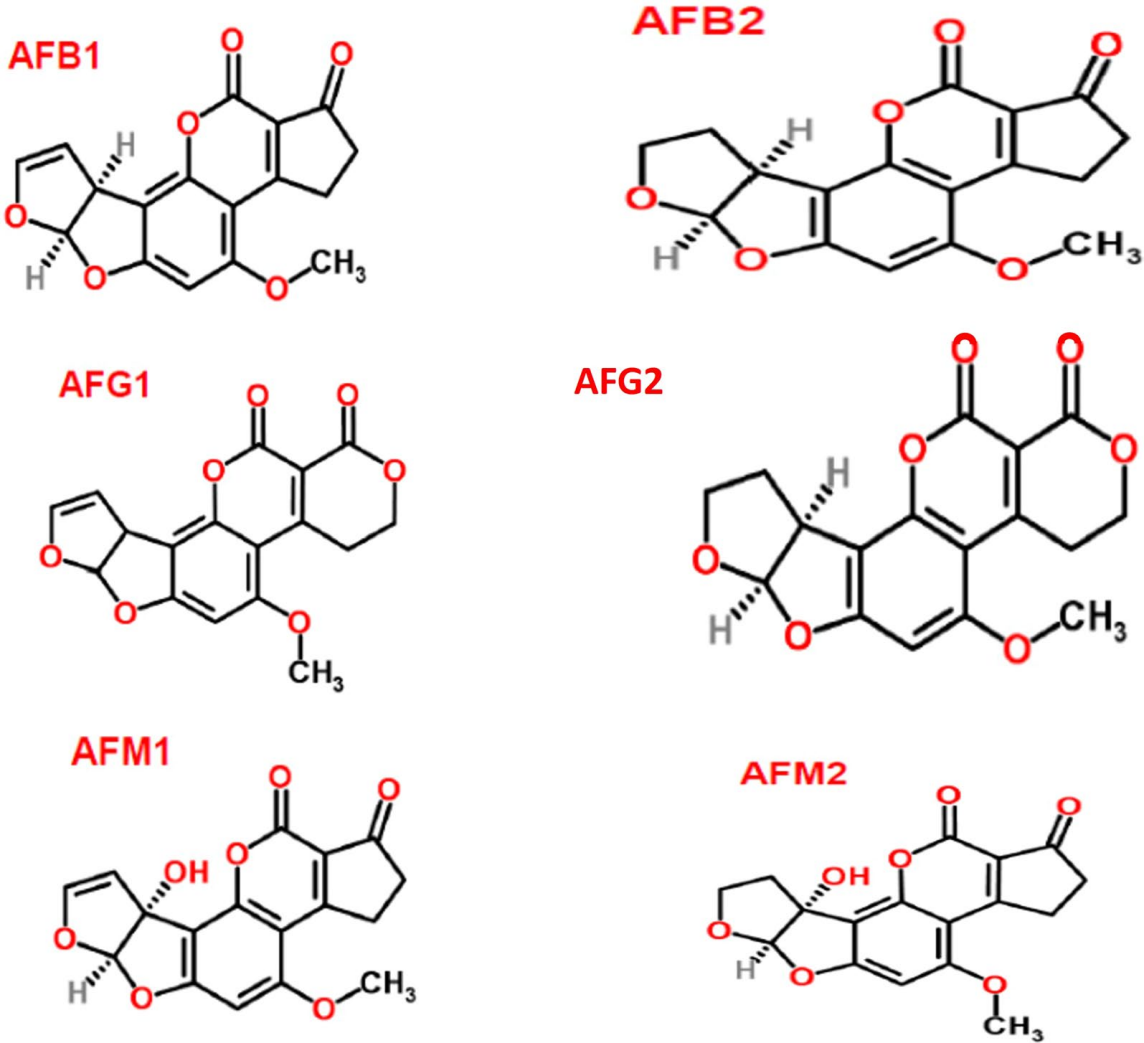
Chemical Structures of Various AFs Forms (structure were created using the ChemSpider|Search and share chemistry)( https://www.chemspider.com/FullSearch.aspx (Accesed on 21 November 2023)

Many *Aspergillus* species, particularly *Aspergillus parasiticus* and *Aspergillus flavus,* are responsible for the production of AFs (Martins et al. 2001). The four main forms of AFs are AFs B1, B2, G1, and G2 (Fig. 1). Total AFs is the name for all AFs taken collectively. AFs are well-known MTs that are produced by molds that thrive in hay, cereals, decomposing plants, and soil. Cereals (such as acha, millet, guinea corn, rice, wheat, sorghum, and corn), tree nuts (such as walnut, coconut, pistachio, and almond), oilseeds (such as sesame, cotton, sunflower, peanut, and soybean seeds), and spices (such as ginger, turmeric, coriander, black pepper, garlic, and chili peppers) are among the crops that are frequently impacted by such moulds. The strongest carcinogen and most harmful toxin known as AFB1 has been directly connected to numerous health issues in various animals, including liver cancer (https://www.who.int/news-room/fact-sheets/detail/mycotoxins) (Accessed on 14 November 2023); Agriopoulou et al. 2020; Martins et al. 2001). Animal dairy and milk products can also include these MTs, especially if the animals were fed contaminated feed (https://www.who.int/news-room/fact-sheets/detail/mycotoxins) (Accessed on 14 November 2023). It is usual to find AFM1, a byproduct of AFB1 detoxication, in dairy products. The primary sources of AFs in feeds are maize, cottonseed, and peanut meal. According to the World Health Organisation (WHO), AFs can cause Acute aflatoxicosis poisoning which can be fatal frequently due to liver damage. It has also been claimed that AFs are genotoxic, meaning they could harm DNA and result in animal cancer. There is enough proof to conclude that AFs cause liver cancer in both humans and animals (Wild & Turner 2002).

### Mechanisms of action of AFs

Numerous studies have been conducted on AFB1, with a spot on the mutagenicity and carcinogenicity of AFs. Due to the double bond at positions 8, and 9, AFB1 is typically metabolized to AFB1-8,9-epoxide, which can attach to biological macromolecules like deoxyribonucleic acid (DNA) in its reactive form (Wild & Turner 2002; Urusov et al. 2015; Anfossi et al. 2016). The primary DNA adduct, a pro-mutagenic lesion called AFB-N7-guanine, frequently results in G-T transversions.

Urine tests show the presence of AFB-N7-guanine, which is utilized as an exposure biomarker in epidemiological research. Because they lack the 8,9 double bond, AFG2 and AFB2 are less physiologically active. AFB1-8,9-epoxide promptly inserts into the DNA in comparison to AFG1, resulting in the development of greater quantities of DNA adduct at any given dose. AFG1 is capable of biological activation to 8,9- epoxide, yet it is not as mutagenic as AFB1 (Agriopoulou et al. 2020; Qiu et al. 2016). Years ago, reports of AFs poisoning in humans were made, yet prior research on the causes seemed to be unclear (Awuchi et al. 2020). The affected individuals initially displayed anorexia, fever, and jaundice after vomiting, which developed into lower extremity edema and ascites. There is proof that people with AFs poisoning exhibit low-grade fever, general malaise, anorexia, stomach discomfort, and tachycardia. Kenya, an East African nation, was the site of an aflatoxicosis incident in 2004 (Lewis et al. 2005; Azziz-Baumgartner et al. 2005). As a result of these outbreaks, hundreds of people died after eating maize infected with AFs. Aflatoxicosis is characterized by severe jaundice of unclear source. Case–control studies on the disease showed that foods from exposed families have much more AFs in them than foods from unexposed families. Examining blood levels of AFs biomarkers revealed significant differences between patients and controls. (Azziz-Baumgartner et al. 2005; McCoy et al. 2008).

Aflatoxin-contaminated maize has been linked to aflatoxicosis and acute hepatitis, and the evidence for this association is strong enough. Most cases of aflatoxicosis are recorded in areas where maize is a common staple grain. It has been investigated how much AFs people consume to get aflatoxicosis and the reasons why (Wild and Gong 2009). Natural AFs are categorized by the International Agency for Research on Cancer (IARC) as Group 1 human carcinogens (https://monographs.iarc.who.int/wp-content/uploads/2018/06/mono82.pdf) (Accessed on 14 November 2023). Moreover, children who live in areas where food contamination is common are exposed to high levels of AFs regularly. Exposure begins during pregnancy and continues during the first few years of life; however, nursing provides some relief from high daily intake. Numerous animal studies have demonstrated that being exposed to AFs has negative impacts on growth (Lombard 2014). Early investigations looked at the connection between AFs exposure and kwashiorkor (Hendrickse et al. 1982). Research also connected the presence of AFs in mothers' blood to considerably lower birth weights in female infants (De Vries et al. 1989).

### Ochratoxin A (OTA)

**Fig. 2:**
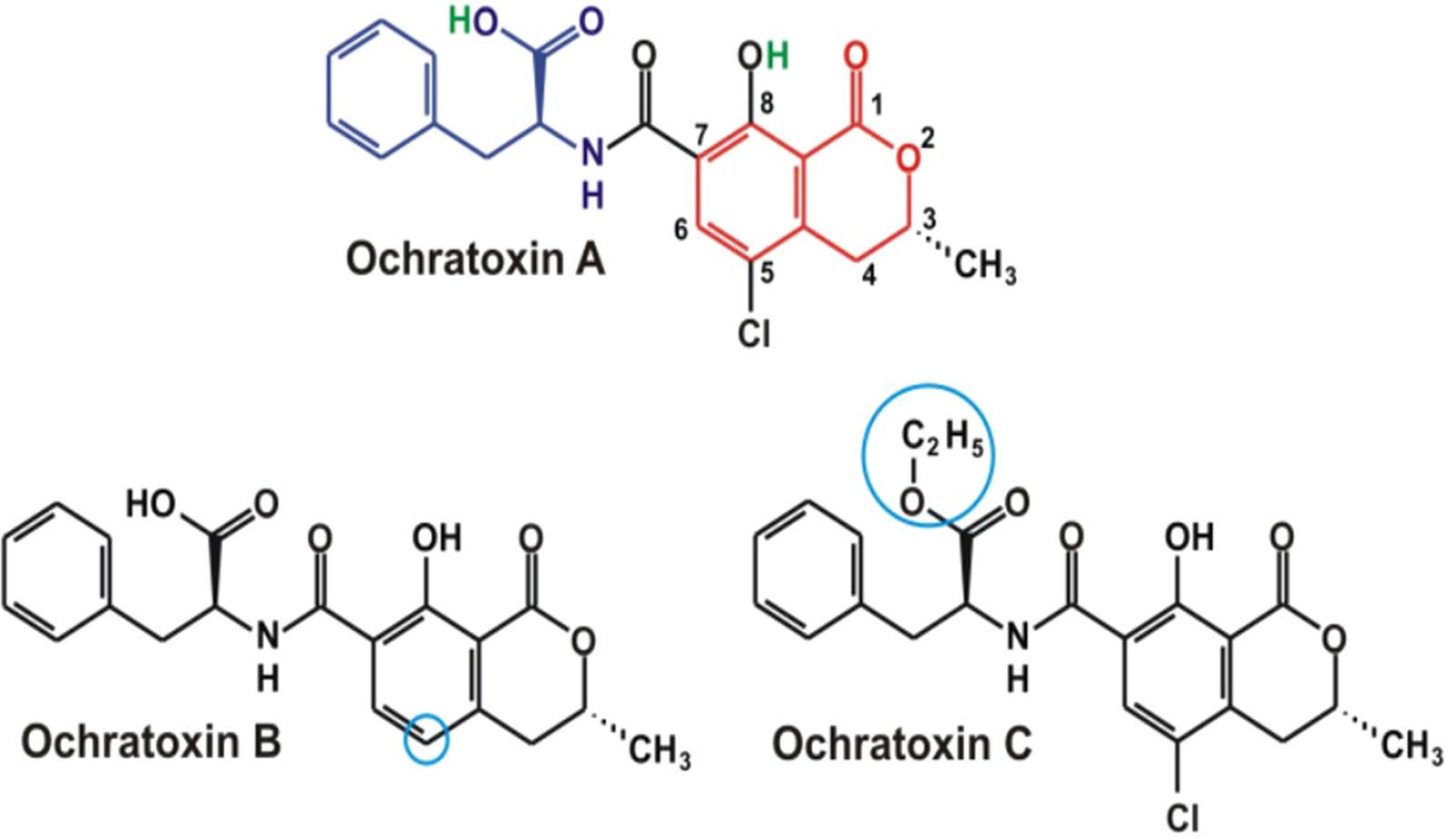
Chemical Structures of Various ochratoxins Forms (structure obtained from ChemSpider|Search and share chemistry) (Accesed on 21 November 2023)

Ochratoxin A (OTA), ochratoxin B (OTB), and ochratoxin C (OTC) are three different MTs known as OTs (Fig. 2). The fungal species *A. niger, A. ochraceus, Aspergillus melleus, Aspergillus sclerotiorum, Aspergillus sulphureus, Penicillium verrucosum, and A. carbonarius* create OTA, which is poisonous. Species of *Aspergillus* and *Penicillium* release all OTs. OTC is OTA’s ethyl ester, whereas OTB is its non-chlorinated version (Bayman and Baker 2006). OTA was initially discovered in the Balkan area (Vrabcheva et al. 2000). Numerous products, including cereals, seeds, coffee, nuts, fruits, dried meat, and alcoholic beverages like wine and beer, are thought to be contaminated by OTA. The primary *Aspergillus* found in vine fruit is *A*. *carbonarius*, which produces harmful byproducts during the production of beverages (Mateo et al. 2007).

### OTA toxicity

Although there has been little research on people due to confounding variables (Bayman and Baker 2006; Mateo et al. 2007) it showed that OTA is a carcinogen and nephrotoxin, directly connected to tumors in the human urinary tract. In poultry and pigs, OTA has been connected to nephropathy. OTA has been linked to the etiology of a number of kidney diseases (Fuchs and Peraica 2005; Marin-Kuan et al. 2008; Pfohl-Leszkowicz and Manderville 2007). Balkan endemic nephropathy (BEN) is a Chronic tubulointerstitial disease that causes irreversible renal failure. Indeed, 15-year research found that BEN is linked to cancer of the upper urothelial tract (Rouprêt et al. 2015). The OTA's toxic action modes are the inhibition of protein synthesis and energy production, the formation of DNA adducts, apoptosis, and oxidative stress induction (Kőszegi and Poór, 2016). Evidence for OTA carcinogenicity primarily comes from research done on experimental an←imals. OTA is carcinogenic to rats and mice according to studies of laboratory, causing kidney cancer in mice and rats and HCC in mice (Bayman and Baker 2006; Mateo et al. 2007).

OTA carcinogenicity's exact mechanism of action is still being investigated. Consuming OTAs has been linked with an increased risk of cancer according to descriptive studies. As stated by the International Agency for Research on Cancer, there is enough data to classify OTA as dangerous for lab animals’ cancer, but not enough to say that it raises the risk of cancer in humans. As a result, OTA is classified by the IARC as Group 2B, potentially carcinogenic to humans (https://www.who.int/news-room/fact-sheets/detail/mycotoxins) (Accessed on 14 November 2023;) (Accessed on 12 November 2023). Other OTA toxicities include kidney lesions in poultry, bone marrow toxicities in mice, GI tract and lymphoid tissue lesions in hamsters, as well as liver and heart lesions in rats and chickens (Pfohl-Leszkowicz & Manderville 2007). Furthermore, recent research has shown that OTA causes autism through an epigenetic mechanism (Mezzelani et al. 2016). Previous research has revealed that OTA causes gut changes in addition to its negative effects on the kidney. Nutrition absorption in the intestine was altered by OTA. In vitro studies revealed that OTA reduced glucose absorption via the SGLT1 transporter (Liew & Mohd-Redzwan 2018).

### Zearalenone (ZEN)

**Fig. 3:**
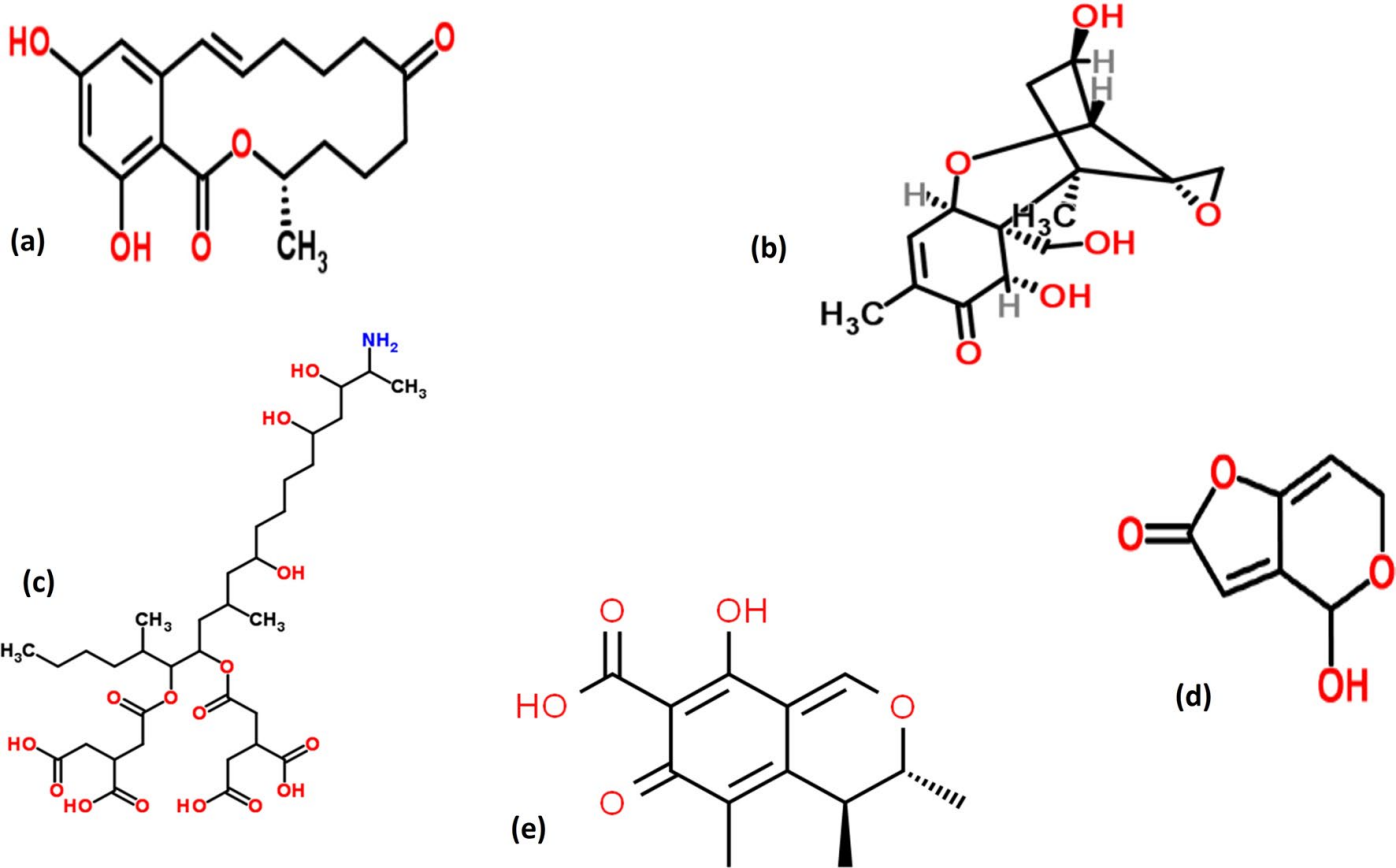
Structural Representation of **a** ZEN **b** DON **c** Fumonisin **d** patulin **e** citrinin (structure obtained from ChemSpider|Search and share chemistry(Accesed on 21 November 2023)

Some *Fusarium* and *Gibberella* species produce ZEN, also named as F-2 mycotoxin (Fig. 3a), which is an estrogenic nonsteroidal metabolite (Bulgaru et al. 2021; Malir et al. 2016). ZEN has been found in oats, almonds, soybeans, and sesame, along with corn, sorghum, wheat, rice, barley, and other grains (Gadzała-Kopciuch et al. 2011).

### ZEN toxicity

Because ZEN resembles naturally occurring estrogens, it has been observed in multiple in vivo experiments to alter the hormonal balance (Abia et al. 2013). Since this MT has a strong affinity for estrogen receptors, it causes fertility and reproductive problems in mammals (El-Sayed et al. 2022). Based on the hormonal mechanism of ZEN and its carcinogenic effect, it can increase the occurrence of pituitary various tumors in mice (Rai et al. 2020**;** https://publications.iarc.fr/74) (Accessed on 15 November 2023). The IARC categorized ZEN as being in Group 3, or not classifiable as human carcinogenic. Additionally, current research indicates that ZEN is metabolized in the liver and has been shown in animal research to have nephrotoxic, immunotoxic, carcinogenic, and hepatotoxic effects (Chatopadhyay et al. 2012). Since this MT is so dangerous to consumer health, the European Union (EU) has set ZEN limits for a diversity of uncooked and processed cereals (20–350 g/kg) (El-Sayed et al. 2022).

Even though its major target is the reproductive organ, adverse effects on the gastrointestinal tract have been documented. When compared to other MTs, the impacts of ZEN ingestion on the GI tract are not as severe. ZEN caused cell death in intestinal epithelial cells without affecting cell integrity. As ZEN can cause hyperkeratotic papillomas in the rat esophageal squamous epithelium stomach, ZEN may contribute to the development of tumors in the gastrointestinal tract. Regions with high MTs contamination are thought to have a higher incidence of esophageal cancer (Richard 2007). In summary, ZEN harms gut health, although no visible histological changes have been observed.

### Deoxynivalenol (DON)

DON (Fig. 3b) is a trichothecene MT that is generated in a variety of cereals like wheat by fungi like *Fusarium graminearum.* Various toxins are released by *fusarium fungus*, which are frequently found in soil. Fumonisins, DON, fumonisol (NIV), T-2, HT-2 toxins, and ZEN are few examples of trichothecenes. (https://www.who.int/news-room/fact-sheets/detail/mycotoxins) (accessed on 14 November 2023).

### DON toxicity

In humans, trichothecenes can be acutely hazardous, causing cutaneous or intestinal mucosal irritation quickly and diarrhea as a result (https://www.who.int/news-room/fact-sheets/detail/mycotoxins) (Accessed on 14 November 2023). DON causes vomiting (hence the name “vomitoxin”), reproductive toxicity, oxidative damage, and digestive problems, but it is not carcinogenic to humans (Ji et al. 2019). DON is categorized as Group 3 by the International Agency for Research on Cancer (IARC) (non-carcinogenic substances) (Ji et al. 2019). DON has been shown to have numerous poisonous effects, such as diarrhea, reduced weight gain, immunotoxicity, teratogenicity, cardiotoxicity, and feed refusal (Chidozie and Pestka 2010; Gray and Pestka 2007). A recent study conducted in 2023 showed that, glycyrrhinic acid and probiotics relieved deoxynivalenol-induced cytotoxicity in intestinal tissues (Xu et al. 2023).

### Fumonisins

MTs called fumonisins are created by the section *Liseola* of the genus *Fusarium*. They structurally resemble sphinganine, the precursor of the sphingolipid backbone (Fig. 3c). The most prevalent fumonisins are types B1, B2, B3, and B4 (FB1, FB2, FB3, and FB4, respectively) (Marasas 2000). There are currently over 28 fumonisins that have been identified and categorized into four classes (A, B, C, and P). (Marasas 2000). Grapes with *Aspergillus welwitschiae* infections were found to have an uncommon class of non-aminated fumonisins in 2015, although their toxicity has not yet been fully determined (Renaud et al. 2015)**.** The majority of fumonisins are found in maize, with smaller amounts in other grains. (https://www.who.int/news-room/fact-sheets/detail/mycotoxins)(accessed on 14 November 2023); https://iris.who.int/bitstream/handle/10665/42448/WHO_TRS_906.pdf;sequence=1)(accessed on 14 November 2023); https://www.who.int/publications/i/item/9789240060760)(accessed on 14 November 2023). Fumonisin has been connected to esophageal cancer in people (Shephard 2012). It also has diverse effects on animals. It has been linked to several disorders, including leukoencephalomalacia in horses and rabbits (Giannitti et al. 2011).

### Patulin

Patulin (Fig. 3d) is released by species of *Aspergillus*, *Penicillium*, and *Paecilomyces*. *Penicillium expansum* is typically found in a wide variety of vegetables, rotting fruits, grains, including rotting maize, apple, peanuts, fig and acha (Awuchi et al. 2019; Moss 2008). Since patulin is known to be destroyed by fermentation, it is not present in apple beverages that are fermented, such as cider. Although patulin has not been proven to cause cancer, it has been shown to impair animal immune systems (Moss 2008). Apples and their juices from diseased fruits are the primary dietary sources of patulin in humans, however, it is also found in numerous grains, fruits, and other foods (https://www.who.int/news-room/fact-sheets/detail/mycotoxins) (Accessed on 14 November 2023).

### Patulin toxicity

Immunological toxicity, spleen damage and toxicity, and toxicity to the liver and kidneys are some of the acute signs of patulin. Gastrointestinal problems, nausea and vomiting are frequently documented in humans. 6-Methylsalicylic Acid is the precursor to patulin; combined, they are acetyl-CoA derivatives, making them polyketides and potential carcinogens (Ahmed Adam et al. 2017). When administered to pregnant mice, patulin has also shown toxicity; both female and male mice died. In addition to damaging the intestine, patulin is carcinogenic, mutagenic, and teratogenic. It also damages cellular DNA in both bacteria and human, which can result in cancer and tumour development (Ahmed Adam et al. 2017; Mahfoud et al. 2002). Even though the IARC has voiced serious concerns about the potential carcinogenicity of patulin, it has assigned the substance to Carcinogenicity Group 3 (Baert et al. 2007). Prior to its discovery as being harmful, patulin was used as an antimicrobial against both Gram-negative and Gram-positive bacteria. As a result, its use as an Antibiotic has been discouraged due to its toxicity (Puel et al. 2010).

### Citrinin

A MT called citrinin (Fig. 3e) was initially discovered in the mould *Penicillium citrinum.* More than 12 *Penicillium* species and multiple *Aspergillus* species have documented cases of it (Bennett and Klich 2003). Additionally, citrinin is produced by several *Monascus species* (Singh and Mehta 2020). MT citrinin is a polyketide. Its natural fluorescence is caused by its conjugated, planar structure; the maximum fluorescence is produced by a nonionized citrinin molecule at pH 2.5 (Singh and Mehta 2020). Citrinin is linked to the yellowed rice illness that has been documented in Japan, according to a study in 2003 by Bennett and Klich. Additionally, it is a nephrotoxin in all studied animal species. Citrinin has been linked to several agricultural grains, including oats, barley, maize, rye, rice, and wheat, as well as foods coloured with the Monascus pigment, although its full effects on humans are still unknown. Citrinin and OTA are said to work together to inhibit RNA synthesis in murine kidneys (Bennett and Klich 2003). Citrinin was identified quantitatively in samples of red fermented rice using high-performance liquid chromatography with fluorescence detection (HPLC-FLD) and LC–MS/MS, and it was found that LC–MS/MS performed better than HPLC-FLD concerning quantification and limit of detection (LOD) (Ji et al. 2015).

### Ergot alkaloids

Ergot alkaloids are poisonous alkaloid combinations that *Claviceps* species, which are popular pathogenic microorganisms of many types of grasses, emit in their sclerotia. Ergotism, often named as St. Anthony’s Fire, is a human disease caused by ingesting ergot sclerotia from infected cereals, typically in the shape of baked bread from polluted flour (Bennett and Klich 2003). Convulsive ergotism, which affects the central nervous system (CNS), and gangrenous ergotism, which is known to damage the blood supply to the extremities, are the two types of ergotism. Ergot alkaloids cause low nerve fever and ergotism and have significant impacts on human fertility (Bhat et al. 2010). Ergotism incidence has been greatly reduced as a human disease, according to Bennett and Klich, but it is still a significant veterinary issue (Bennett and Klich 2003). Additional file 1: Table S1 is a summary of the different types of MT, Predominant Food Sources, Toxicity Levels, IARC Carcinogenicity Classification, and Regulatory Limits in the US and EU. The different factors affecting MT production are summarized in Additional file 1: Table S2.

### Analysis of MTs

More reliable analytical techniques for MTs determination are desperately needed, as the EU and other developed countries have reduced the restriction limits of MTs in foods and feeds (EC466 2001; EC472 2002). Currently, the most often used analytical techniques are confirmatory quantification and fast screening approaches. Trichothecenes in food and several other MTs in feed are being studied, and standardized procedures for AFs (EN12955 1999; EN14123 2001), OTA (EN14132 2003), fumonisins (EN13585 2001; EN14352 2004), and patulin (EN14177 2003) in diverse foods are available. A comprehensive set of official MTs analysis methods has been published in previous studies (Rahmani et al. 2009). The International Official Procedures of Analysis of the AOAC 991.31(Association of Agriculture and Culture) includes approved analytical techniques for determining the presence of MTs in food and feed (Rai et al. 2020)**.** MTs levels in food samples are often determined using procedures that involve the sampling, homogenization, extraction, cleanup, and ultimately detection and quantification, which are carried out using a variety of instrumental and non-instrumental approaches (Pereira et al. 2014; Shephard 2016; Whitaker 2003).. Biological degradation as a method of analysis proved to be more effective, specialized, and environmentally friendly (Xia et al. 2022).

### Sampling

Among environmental factors, humidity and temperature have the greatest effect on mycotoxigenic fungi to produce MTs. In terms of Optimal storage procedures, temperature, humidity, and moisture content in the warehouse are critical factors for mould growth and MTs production (Agriopoulou et al. 2020). MTs are generated in isolated areas and are not uniformly distributed in commodities that are stored. Furthermore, because of its heterogeneity, it is difficult to collect representative samples. By making the sample size larger, degree of crushing, and number of aliquots quantified, the inconsistency associated with MTs analyses is reduced (Whitaker 2003). EC has defined sample collection requirements as well as performance criteria for analytical techniques (Elkenany and Awad 2020). The method used to sample grains and grains products for lots under 50 tonnes, for instance, calls for the employment of a sampling plan and incremental samples of 10 to 100, depending on the weight, for an aggregate sample of 1 to 10 kg (https://food.ec.europa.eu/system/files/2016-10/cs_contaminants_sampling_guidance-sampling-final_en.pdf#:~:text=Commission%20Regulation%20%28EC%29%20No%20401%2F2006%20of%2023%20February,for%20the%20control%20of%20mycotoxins%20in%20various%20foodstuffs). (Accessed on 25 september 2023).

### Sample preparation (grinding and mixing)

The sample should be homogenised and milled to a final particle size of around 500 µm opening size to speed up the chemical reaction process of extraction and improve the likelihood that the MTs will be detected (Nakhjavan et al. 2020). The sample should be blended once homogeneity has been achieved. slurry mixing yields lowest variation ratio. (Spanjer et al. 2006).

### Extraction and purification (clean up)

#### Extraction

The initial step in sample preparation is MTs extraction from the sample, which is succeeded by cleanup techniques to improve the specificity and sensitivity of a particular detection method (krska 1998). Three main considerations often determine the choice of extraction and cleanup procedures for MTs from food samples: the chemical makeup of the MTs, the makeup of the food matrix, and the intended technique of detection (Ridgway et al. 2012). The QuEChERS (Quick, Easy, Cheap, Effective, Rugged, and Safe) procedure is an extraction using acetonitrile–water, followed by the induction of liquid–liquid partitioning with adding inorganic salts followed by dispersive solid phase extraction to remove additional matrix components from the organic phase (González-Jartín et al. 2019). Another extraction technique, called liquid–liquid extraction (LLE), depends on the differing solubilities of toxins in aqueous and immiscible organic layers (Turner et al. 2009). The extraction of MTs from solid matrices of varied consistencies can be accomplished easily using the liquid–solid extraction (SLE) technique (Xie et al. 2016). Pressurised liquid extraction (PLE), commonly referred to as accelerated solvent extraction (ASE), is the similar process to solvent-free extraction (SLE), but it is carried out at higher temperature and pressure in a pressure-resistant vessel (Rico-Yuste et al. 2018). These techniques use ordinary solvents at high pressures (1500–2000 psi) and temperatures (100–180 °C) to enhance the extraction of analytes from the matrix **(**Razzazi-Fazeli and Reiter 2011). Supercritical Fluid Extraction (SFE) is another technique. By using supercritical CO2, SFE can reduce or eliminate the need of organic solvents. The SFE process is primarily used to extract non-polar chemical compounds (Xie et al. 2016).

#### Clean-up

After extraction, it's critical to further clean up the extract to lessen matrix impacts and get rid of everything that might get in the way of the next MT detection. The extract's purification improves the extract's specificity and sensitivity, which raises the accuracy and precision of measurement. Immunoaffinity columns (IAC) and solid phase extraction (SPE), which are quick, effective, repeatable, and have a broad spectrum of selectivity, are the two techniques most frequently employed for MTs cleanup (Alshannaq & Yu 2017; Razzazi-Fazeli & Reiter 2011)**.** The SPE method involves the solid absorbents and capture the MTs (Huertas-Pérez et al. 2017). SPE is a quick, effective, and repeatable technology, but it has significant drawbacks, such as the difficulty to identify all mycotoxins with a single cartridge. Additionally, several factors, including the solvent type used or the ionic strength and pH of the sample, might have an impact on efficiency (Pereira et al. 2014).

Monoclonal antibodies are employed in the case of IAC to identify specific MTs. Particular antibodies on the column bind the target MT in the extract as the sample flows through the column. Pure methanol or acetonitrile is used to elute the MTs from the IAC for further detection while water-soluble contaminants are also eliminated during column washing. IACs are a highly sensitive and selective purification method that can be used to identify MTs. Because of the specificity of the antibodies, it is also a solvent-saving and easy-to-use technique (Liu et al. 2018). However, this strategy has significant drawbacks. MTs can only be absorbed by columns to a certain extent; if the sample's MTs content exceeds this limit, the MTs cannot be efficiently captured and bound, leading to incorrect results. Furthermore, the matrix's many components may conflict with the antibodies (Castegnaro et al. 2006). Moreover, the organic solvents have another disadvantage as they might denature the antibodies, and has very high operational costs (Liu et al. 2018).

### Conventional techniques used in detection and analysis of MTs

Numerous techniques have been tried and tested to determine the presence of MTs in food and feed since the first MTs were discovered (Le et al. 2021). The employment of several distinct chromatography types, including High-performance liquid chromatography (HPLC) and thin-layer chromatography (TLC) in combination with diverse detectors including UV, fluorescence, and diode array, is what primarily accounts for the supremacy of chromatographic techniques. MTs detection has also made extensive use of Liquid chromatography-tandem mass spectrometry (LC–MS/MS) and gas chromatography-tandem mass spectrometry (GC–MS/MS) (Turner et al. 2015). Immunoassay techniques, such as (ELISA) enzyme-linked immunosorbent assay, (Hendrickson et al. 2018) and (LFIA) lateral flow immunoassay also (Lattanzio et al. 2019) are used when a quick mycotoxin detection is necessary. A recent study conducted by Boshra et al. (2023) revealed no significant differences were determined between ELISA and immunoaffinity fluorometric analysis. They can substitute for each other whenever necessary. However, significant differences were detected upon analyzing different food categories, highlighting the urgent need for more specific, rapid and accurate detection methods that can cover all food categories whenever possible (Boshra et al. 2023).

### Chromatography techniques

#### Thin layer chromotography (TLC)

TLC is a well-known method of MT detection that allows for the cost-effective screening of several samples (Yang et al. 2014). TLC consists of a stationary phase consisting of cellulose, silica, or immobilized alumina on an inert matrix made of glass or plastic. Methanol, acetonitrile, and water mixes make up the mobile phase, which transports the sample in the solid stationary phase (Wacoo et al. 2014). It is crucial in the investigation of several MTs due to its simplicity, low costs and luminous spots under UV light. This method was created for MTs qualitative (Abrunhosa et al. 2001) and quantitative analysis (Andrade et al. 2013)**.** However, due to TL’s weak accuracy and sensitivity, quantification is quite difficult (Singh & Mehta 2020). Additionally, one of the primary criteria is sample preparation and the kind of cleanup method, that heavily relies on the characteristics and MT type (Yang et al. 2014).

#### Liquid chromatography (LC)

The LC methods have been created to get over some of the TLC technique's drawbacks, such as the limited plate height or effects of temperature and humidity (Singh and Mehta 2020). A mobile phase and an analytical column are utilized to separate the analytes from the matrix components, and for high polarity, non-volatile, and thermally labile MTs, LC is also utilized as a separation and determination method. This is true regardless of their biological activity and chemistry (Yang et al. 2020). According to the physical and chemical makeup of the MTs, the analysis of MTs mainly depends on HPLC with various adsorbents. Most of the detection procedures for MTs are relatively similar. The most popular HPLC detectors are fluorescent (FLD) or UV–visible (UV) ones, which depend on the molecules having a chromophore but also on MS (single mass spectrometry, and tandem MS (MS/MS) (Turner et al. 2009). Some MTs such AFs and OTA already have a natural fluorescence and can be found in HPLC-FLD without further testing. For the detection of OTA in diverse matrices, like rice, HPLC-FLD is most frequently utilized (Zinedine et al. 2007). Derivatization is required for other varieties of MTs, like fumonisin B1 (FB1), which have no chromophores in their composition (Zhang et al. 2018a, b). The portability, practical concerns depending on the matrix impact, sample preparation and type, as well as the calibration, are the primary drawbacks of the HPLC technique (Singh & Mehta 2020). Over the past two decades, there has been a substantial growth in the usage of LC–MS/MS for the detection of low molecular weight pollutants and residues. Better reliability and sensitivity are offered by MS/MS when combined with LC. Because of this, LC–MS/MS is an excellent standard instrument for addressing the analytical issues in food and feed safety chemical analysis, both in research and in a commercial study (Malachová et al. 2018). Compared to conventional procedures employing conventional detectors, LC–MS/MS offers excellent selectivity and sensitivity, greater assurance of analyte identification, and a larger choice of matrices (Pascale et al. 2019).

#### Gas chromatography (GC)

The differential analytes partitioning between the two GC column phases is essential for GC. Between the stationary and mobile phases, the sample's numerous chemical components are distributed. Utilizing a flame ionization detector (FID), a mass spectrometer, or an electron capture detector (ECD), volatile compounds are found following the separation procedure (Singh and Mehta 2020). Due to the minimal volatility and strong polarity of the analytes, GC is not frequently utilized in the analysis of MTs. Additionally, the derivatization process is necessary for their transformation into volatile derivatives (Alshannaq and Yu 2017). However, volatile MTs like trichothecenes (TCTC) and patulin have been identified and quantified using gas chromatography (GC) in conjunction with flame ionization (FID), electron capture (ECD), or MS detectors (Pereira et al. 2014). The method can be derivatized to a chemical that is volatile enough to be applied to gas chromatography and is very sensitive and specific to MTs. Column obstruction, swaying consequences, cross-contamination from previous samples, and nonlinearity of calibration curves in specific detector types are the main issues in MTs GC analysis (Singh & Mehta 2020).

#### Enzyme-linked immunosorbent assay (ELISA)

Immunochemical approaches, like ELISA, are quick and easy screening procedures for the on-site MTs analysis together with the sensitive but difficult and expensive techniques of chromatography (Al-Jaal et al. 2019). ELISA is easy to use, allows for simultaneous examination of numerous samples, and has accurate detection (Urusov et al. 2010). In comparison to chromatographic techniques like HPLC or TLC, it requires less sample volume, fewer clean-up steps and is a high-throughput test (Singh & Mehta 2020). The antigen–antibody complex's interaction with chromogenic substrates serves as the basis for the test. By using spectrophotometric analysis, the quantitative outcome is obtained (Li et al. 2009). This method does, however, have evident disadvantages. The antibodies can react with elements that share similar chemical moieties (Thway & Salimi-Moosavi 2014). Furthermore, inadequate ELISA validation limits the method to the media for which they have accepted validation (Omar et al. 2020).

#### Lateral flow immunoassay (LFIA)

As a signal reagent, a labeled antibody is utilized in the membrane-based immunoassay known as LFIA, also known as the immunochromatographic strip test (Song et al. 2014). Capillary beds, which resemble porous pieces of paper, drive the analyte during the test, and particular elements of recognition bind moieties adsorbed on the surface of the membrane (Anfossi et al. 2013). Signal labels have a major impact on LFIA accuracy. Gold nanoparticles (GNPs) have historically been the most popular label for producing visual signals (Li et al. 2019). Commercially available LFDs are accessible for the identification of OTA, ZEN, DON, T-2 toxin, and AFs (Krska & Molinelli 2009). However, because of several issues with the sensitivity and dependability of various matrices, their use in the field is limited (Goryacheva et al. 2007).

### Limitations and current challenges of the conventional detection methods

Although numerous conventional techniques including different chromatographic methods, ELISA, and immunoaffinity methods, have been extensively employed for the detection of various MTs in food. However, they still have many drawbacks and limitations such as the need for accurate and very long procedures for sample preparation (including, grinding, mixing, and ensuring homogenization), extraction, and clean up which are considered very tedious processes in addition to the extensive use of solvents, need of well-trained personnel as well as high cost of analysis. Because of heterogeneity of the tested samples, it is difficult to collect representative samples. Therefore, by making the sample size larger, degree of crushing, and number of aliquots quantified, the inconsistency associated with MTs analyses in food is reduced (Whitaker 2003). Moreover, organic solvents have another disadvantage as they might denature the antibodies in the case of ELISA and Immunoaffinity analysis, and besides the very high operational costs (Liu et al. 2018). All such factors encourage researchers worldwide to find and examine novel approaches to circumvent the respective drawbacks of the conventional methods of analysis.

### Novel technologies of mycotoxins analysis and detection

#### Biosensors

Typically, biosensors include a transducer that transforms biological signals into electrical signals, along with a biological or sensory element with a biological basis to identify bio-analytes (Perumal and Hashim 2014). MTs detection can be carried out using a variety of transducers, including optical (fluorescence and surface plasmon resonance-SPR), electrochemical (potentiometric, amperometric, and impedimetric), and piezoelectric (quartz crystal microbalance-QCM) ones (Santana et al. 2019). Cells, peptides, enzymes, antibodies, and nucleic acids are well-known materials, but other bioinspired components can also be used, such as molecules imprinted polymers (MIPs), aptamers, and recombinant antibodies (Malekzad et al. 2017). Additionally, a wide range of QDs, metal nanoparticles, nanofibers, and carbon nanotubes (CNTs) are used in the biosensors to increase their sensitivity because of their physicochemical properties, biocompatibility, and a high surface volume ratio (Doria et al. 2012). One significant privilege of biosensors over other rapid screening strip tests is their possibility for recycling use. Surface plasmon biosensor chips with DON immobilized can be reused more than 500 folds without experiencing significant activity reduction (Tüdös et al. 2003). Most biosensor processes still require sample cleanup, even though several formats for biosensors could be helpful in MTs analysis. Additionally, the equipment is unable to do numerous analyte studies simultaneously (Logrieco et al. 2005).

### Electronic nose

An electronic nose, often known as a “e-nose,” is made up of a variety of general-purpose chemical detectors that can pick up a variety of volatile organic compounds (VOCs) and identify the toxic fungi’s qualitative volatile fingerprints. Finding a fingerprint comes after odor identification provides a pattern recognition system’s early classification of the generated metabolites (Camardo et al. 2021). E-nose technology depends on recognizing particular VOCs connected to the fungi growth on grains to detect fungal infections. A relationship between VOCs and the amount of MTs in food can be seen, and this relationship is influenced by the proliferation and metabolic pattern of mycotoxigenic fungal species (Ottoboni et al. 2018). The e-nose has been utilised well to find OTA in the dry-cured pork (Lippolis et al. 2016), AFs and fumonisins in maize (Ottoboni et al. 2018), and DON in wheat bran (Lippolis et al. 2018). The measurement of low quantities of MTs in food samples must be optimized to accomplish widespread use of e-nose for the identification of MTs. A further issue with e-nose detection is that the bulk of MTs are non-volatile chemical substances (Alshannaq & Yu 2017).

#### Fluorescent polarization

The principle behind fluorescent polarization (FP) immunoassay is that the tracer and the analyte (fluorophore-labeled analyte) compete for antibody-binding sites. The fluorescence polarization value is raised by the tracer's rotation due to the tracer's binding to the antibody. The value of polarization has an inverse relationship to the analyte concentration because the amount of bound tracer has an inverse relationship to the concentration of free analyte in the sample (Valenzano et al. 2014). Some immunoassay procedures, such as ELISA, demand that the analyte be separated from antibody-bound analyte or washed several times. The pre-analytical processes that consume time are not required with the FP approach (Huang et al. 2020). FP immunoassay has been used to identify a variety of MTs in food products, including ZEN in corn (Zhang et al. 2017), DON in wheat-based products (Lippolis et al. 2006), AFB1 in maize (Zhang et al. 2018a, b), and OTA in rice (Huang et al. 2020). Compared to HPLC, the FP method has lower accuracy and sensitivity. This is most likely caused by antibodies' cross-reactivity with food matrix components and other fungal metabolites (Alshannaq and Yu 2017).

#### Capillary electrophoresis

Using fluorescence or UV absorbance, capillary electrophoresis (CE) separates various components according to electrochemical potential. Small volumes of solvents and buffers are needed for this approach, which has the particular advantage of producing only small amounts of waste (Shephard 2008). Numerous MTs have been distinguished by CE, including AFs, DON, fumonisins, OTA, and ZEN (Maragos &Appell 2007). However, as only small sample quantities can be evaluated, this approach lacks sensitivity (Maragos 1998). ZEN in maize has recently been analyzed using CE combined with cyclodextrin-enhanced fluorescence, which has a 5 ng/g detection limit (Maragos &Appell 2007).

#### Infrared spectroscopy

Optical non-destructive and Fast methods for MTs detection in grains include principal component analysis (PCA) and infrared (IR) analyzers for identification and quantitative determination of MTs without preparation of sample**.** These procedures have the advantages of being simple to use, not needing the use of chemicals, extraction or sample preparation and having quick results (Pettersson and Aberg 2003). Although the two methods face difficulties, including the non-homogeneous distribution of MTs within the food matrix, the particle size distribution of ground grains, and the detection limits of the method, more research is required to fully realize IR spectroscopy's potential for detecting various MTs (Shepherd, 2008).

### The aggregation-induced emission

A collection of fluorescent dyes shines dimly in the condition of diluted solution, but their fluorescence is noticeably amplified in the state of aggregation due to a photophysical phenomenon known as aggregation-induced emission (AIE) (Zhu et al. 2019). One possible explanation for the high fluorescence of dyes in the aggregate state could be limited intramolecular rotations (Li et al. 2018). AIE dyes such as 9,10-distyryllanthracene (DSA), silacyclopentadiene (silole), tetraphenylethene (TPE), and its derivatives exhibit high emission of fluorescence in the aggregate states (Wang and Liu 2018). Aptasensor (biosensor) based on AIE dye, has been created effectively for OTA detection in wine and coffee (Zhu et al. 2019). Table 1 summarizes different types of technologies and which technologies can be applied best in different circumstances in terms of sample material, sample condition cost-effectiveness and comparison of sensitivity for these methods.

**Table 1:** Comparative analysis of mycotoxin detection technologies: Suitability across aample materials, conditions, cost considerations and sensitivity of each method

Technology type	Suitability across sample materials, conditions, and cost considerations	Sensitivity of each method	References
TLC	Cost-effective and can be used for the screening of several samples. needs sample preparation	Low sensitivity and poor accuracy	(Yang et al. 2014; Singh and Mehta 2020)
LC	For high polarity, non-volatile, and thermally labile MTs	LC–MS/MS offers excellent selectivity and sensitivity, greater assurance of analyte identification, and can be used for the detection of multi-mycotoxins	(Yang et al. 2020; Pascale et al. 2019)
GC	Due to the minimal volatility and strong polarity of the analytes, GC is not frequently utilized in the analysis of MTs. Additionally, the derivatization Process is necessary for their transformation into volatile derivatives. volatile MTs like TCTC and patulin have been identified and quantified using gas chromatography (GC) in conjunction with flame ionization (FID), electron capture (ECD), or MS detector	The method can be derivatized to a chemical that is volatile enough to be applied to gas chromatography and is very Sensitive and specific to MTs	(Alshannaq and Yu 2017; Pereira et al. 2014; Singh and Mehta 2020)
ELISA	Quick and easy screening procedures for the on-site MTs analysis, accurate detection, Effective for routine monitoring, especially in resource-limited settings. In comparison to chromatographic techniques like HPLC or TLC, it requires less sample volume, fewer clean-up steps and is a high-throughput test	Cross reactivity (less specificity and sensitivity)	(Al-Jaal et al. 2019; Urusov et al. 2010; Singh and Mehta 2020; Thway a Salimi-Moosavi 2014)
LFIA	Quick results, economic and is suitable for large-scale on-site screening. sample clean-up can be neglected. It is used for the identification of OTA, ZEN, DON, T-2 toxin, and AFs	Less sensitive	(Krska and Molinelli 2009; Goryacheva et al. 2007; Liu et al. 2020)
Biosensors	Rapid screening strip tests, easy and inexpensive sample analysis, reproducibility, stability, and on-site testing of samples, possible for recycling use. Require sample clean up	High sensitivity and real-time analysis are the main advantages of optical biosensors	(Tüdös et al. 2003; Pirinçci et al. 2018; Logrieco et al. 2005; Chen and Wang 2020)
Electronic nose	It can pick up a variety of volatile organic compounds (VOCs). It can be used to detect OTA in dry-cured pork, AFs, and fumonisins in maize, and DON in wheat bran Apples, oranges, strawberries, and peaches are some fruits in which the application of this technique has been successfully implemented for the detection of fungi that produce mycotoxins	Unique fingerprint for each food, characteristic of its taste and aroma. Less sensitive to low quantities of MTs	(Camardo et al. 2021; Lippolis et al. 2016, 2018; Ottoboni et al. 2018; Jia et al. 2019)
Infrared spectroscopy	No need for preparation of samples	Less sensitive compared to other techniques	(Pettersson and Aberg 2003)
Fluorescent polarization	Used to identify ZEN in corn, DON in wheat-based products, AFB1 in maize), and OTA in rice Does not require preanalytical steps like washing many times as done in ELISA	Comparatively to HPLC, the FP method has lower accuracy and sensitivity (antibodies' cross-reactivity with food matrix components)	(Zhang et al. 2017; Lippolis et al. 2006; Zhang et al. 2018a, b; Huang et al. 2020; Alshannaq and Yu 2017)
Capillary electrophoresis	Only small sample quantities can be evaluated	Lacks sensitivity	(Maragos 1998)
Aggregation induced emission	The on-site detection of food contaminations and the simple operation make the application of AIE dyes very effective	Highly sensitive AIE dyes showed high affinity to aptamers and fluoresce through the process of dye aggregation	(Zhu et al. 2019)

### Current challengs and future directions

Several novel techniques that have been created and may be helpful in MTs detection have been developed in addition to the traditional techniques mentioned above. However, outside of the study fields, these techniques have not been extensively used and have limited utility. Additionally, they need additional validation and verification from reputable organizations like the European Standardization Committee (EN), International Organization for Standardization (ISO), or Association of Official Analytical Chemists (AOAC) (Alshannaq & Yu 2017).

In conclusion, in food and feed all over the world, MTs are unpredictable pollutants. These low molecular weight substances constitute a significant risk to human and animal health, raise questions about food safety, and cause the agriculture sector to suffer enormous financial losses. Although Numerous conventional techniques including different chromatographic methods, ELISA and immunoaffinity methods, have been extensively employed for the detection of various MTs in food. However, they still have many drawbacks and some limitations such as tedious sampling, extensive use of solvents, need of well-trained personnel as well as high cost of analysis. Various innovative approaches have been recently studied to bypass the disadvantages of conventional methods; however, they are still not widely used and have limited utility. Additionally, they need additional validation and verification from reputable and standard organizations and committees.

### Supplementary Information

**Additional file 1:** **Table S1**. Comprehensive Overview of Mycotoxins: Their Varied types and forms Predominant Food Sources, Toxicity Levels, IARC Carcinogenicity Classification, and Regulatory Limits in the US and EU. **Table S2**. Contributing Factors to MTs Production: Effects and Required Conditions.

## Data Availability

All data generated or analyzed during this study are included in this published article and supplementary file.

## Abbreviations

ASE: Accelerated solvent extraction
Afs: Aflatoxins
ATs: *Alternaria* toxins
AOAC: Association of Official Analytical Chemists
CNTs: Carbon nanotubes
CNS: Central nervous system
DON: Deoxynivalenol
ECD: Electron capture detector
Ens: Enniatins
ELISA: Enzyme-linked immunosorbent assay
Eas: Ergot alkaloids
EC: European Commission
FID: Flame ionization detector
FAO: Food and Agriculture Organisation of the United Nations
FBs: Fumonisins
GC: Gas Chromatography
GC–MS/MS: Gas chromatography-tandem mass spectrometry
HCC: Hepatocellular carcinoma
HPLC: High-performance liquid chromatography
IAC: Immunoaffinity column
IARC: International Agency for Research on Cancer
ISO: International Organization for Standardization
LFIA: Lateral flow immunoassay
LOD: Limit of detection
SLE: Liquid–solid extraction
LC–MS/MS: Liquid chromatography-tandem mass spectrometry
MIPs: Molecules imprinted polymers
MTs: Mycotoxins
OTA: Ochratoxin A
PAT: Patulin
PLE: Pressurised liquid extraction
SLE: Solvent-free extraction
TLC: Thin-layer chromatography
TCTC: Trichothecenes
VOCs: Volatile organic compounds
WHO: World Health Organisation
ZEN: Zearalenone
