# Supplementary material for: Innovative approaches for mycotoxin detection in various food categories
Source: AMB Express. 2024 Jan 12;14:7. doi: 10.1186/s13568-024-01662-y (PMC10786816; doi:10.1186/s13568-024-01662-y)
Supplement: Supplementary file 1 — Additional file 1: Table S1. Comprehensive Overview of Mycotoxins: Their Varied types and forms Predominant Food Sources, Toxicity Levels, IARC Carcinogenicity Classification, and Regulatory Limits in the US and EU. Table S2. Contributing Factors to MTs Production: Effects and Required Conditions. [file 13568_2024_1662_MOESM1_ESM.docx]

**Innovative Approaches for Mycotoxin Detection in Various Food Categories**

Marina H. Boshra ^1^, Ghadir S. El-Housseiny ^2^, Mohammed M.S. Farag ^3,4^, Khaled M. Aboshanab ^2^*

1 Department of Mycotoxins, Central Public Health Laboratories (CPHL), Ministry of Health, Cairo, Egypt. [merohos_2010@yahoo.com](mailto:merohos_2010@yahoo.com)

2 Department of Microbiology and Immunology, Faculty of Pharmacy, Ain Shams University, Cairo 11566, Egypt; [ghadir.elhossaieny@pharma.asu.edu.eg](mailto:ghadir.elhossaieny@pharma.asu.edu.eg)

3 Botany and Microbiology Department, Faculty of Science, Al-Azhar University, Cairo 11884, Egypt, [mohamed.farag@azhar.edu.eg](mailto:mohamed.farag@azhar.edu.eg)

4 Armed Forces College of Medicine (AFCM), Cairo, Egypt

***Corresponding Author**: **Khaled M. Aboshanab (PhD)**

Address: Department of Microbiology and Immunology, Faculty of Pharmacy, Ain Shams University, organization of African unity St. PO: 11566, Abbassia, Cairo, Egypt.

E-mail: [aboshanab2012@pharma.asu.edu.eg](mailto:aboshanab2012@pharma.asu.edu.eg)

Tel: (202)28429040

Mobile: (002)01007582620

Fax: (202)24051107

<https://orcid.org/0000-0002-7608-850X>

**Table S1.** Comprehensive Overview of Mycotoxins: Their Varied types and forms Predominant Food Sources, Toxicity Levels, IARC Carcinogenicity Classification, and Regulatory Limits in the US and EU".

| **MTs types** | **MTs-Producing Species** | **Predominant Food Sources** | **Toxicity levels** | **US FDA** **(µg/kg)** | **EU (EC 2006) (µg/kg)** | **IARC Classification** | **References** |
| --- | --- | --- | --- | --- | --- | --- | --- |
| AFs (B1, B2, G1, G2, M1, M2) | Numerous *Aspergillus* species, particularly *Aspergillus parasiticus and Aspergillus flavus* | Tree nuts (almond, pistachio, coconut, walnut), Cereals (wheat, sorghum, rice, acha, millet, guinea corn, corn, etc.), Spices (garlic, black pepper, coriander, turmeric, ginger, and chilli peppers), and Oilseeds (peanut, sunflower, cotton seeds, soybean, and sesame). | The majority of AFs are genotoxic, hepatotoxic, mutagenic , immunosupressive etc. and can stunt children's growth. The most dangerous toxin is AFB1, which has been directly related to various illnesses in both humans and animals, including liver cancer. The general assessment of AFs detrimental effects on human and animal health is aided by knowledge of the production of mutations, DNA damage, and metabolism in those who have consumed them. | 20 for total | 2–12 for B1  4–15 for total | Group 1: carcinogenic to human | ([Mycotoxins (who.int)](https://www.who.int/news-room/fact-sheets/detail/mycotoxins) (accessed on 14 september 2023); Lombard, 2014;  Mishra & Das, 2003; Streit et al., 2012; [untitled (europa.eu)](https://eur-lex.europa.eu/legal-content/EN/TXT/PDF/?uri=CELEX:02006R1881-20140701&from=EN) (accessed on 12 september 2023); [Microsoft Word - Preamble_updated2015 (who.int)](https://monographs.iarc.who.int/wp-content/uploads/2018/06/CurrentPreamble.pdf)(accessed on 20 september 2023); [Agents Classified by the IARC Monographs, Volumes 1–134 – IARC Monographs on the Identification of Carcinogenic Hazards to Humans (who.int)](https://monographs.iarc.who.int/agents-classified-by-the-iarc/) (accessed on 20 september 2023) |
| OTs (OTA, OTB, OTC) | *Aspergillus pseudoelegans, A. alutaceus, A. alliaceus, A. auricomus, A. glaucus, A. niger, A. carbonarius, A. melleus, A. albertensis, A. citricus, and A. flocculosus .* | cereals (especially wheat and barley) and their derivatives; meat (especially pig, from animals that ate contaminated grains); dried fruits from vines; spices; licorice; coffee beans; wine; grape juice; roots; etc. | OTA has been associated with tumours in human urinary tract and is nephrotoxic, hepatotoxic, teratogenic, genotoxic, immunosuppressive, and carcinogenic . Additionally, it affects ATP production, protein synthesis, and peptidases' ability to detoxify the body. | Not set | 2–10 | Group 2B: possible carcinogenic to human | (Bhat et al., 2010; Zinedine et al., 2007; Bayman & Baker, 2006; Mateo et al., 2007;  [untitled (europa.eu)](https://eur-lex.europa.eu/legal-content/EN/TXT/PDF/?uri=CELEX:02006R1881-20140701&from=EN) (accessed on 12 september 2023); [Agents Classified by the IARC Monographs, Volumes 1–134 – IARC Monographs on the Identification of Carcinogenic Hazards to Humans (who.int)](https://monographs.iarc.who.int/agents-classified-by-the-iarc/) (accessed on 20 september 2023); [IARC Publications Website - Overall Evaluations of Carcinogenicity: An Updating of IARC Monographs Volumes 1–42](https://publications.iarc.fr/139) (accessed on 20 september 2023); [Cover82.qxd (who.int)](https://monographs.iarc.who.int/wp-content/uploads/2018/06/mono82.pdf)(accessed on 14 september 2023). |
| ZEN, also known as F-2 mycotoxin | *Fusarium sporotrichioides, F. culmorum, F. equiseti, and F. graminearum.* | grains (especially rye, oats, barley, wheat, corn, rice, millet, sorghum, etc.), etc. | ZEN has estrogenic activity, which results in feminization of males, infertility, vulvar edoema, vaginal prolapse, and female hypertrophy. In mice, zearalenone can enhance the likelihood of pituitary tumours and liver cells, in keeping with its hormonal route of carcinogenic activities. | Not set | 20–100 | Group 3: not carcinogenic to human | (Bulgaru et al., 2021; Rogowska et al., 2019; Zain, 2011; [IARC Publications Website - Overall Evaluations of Carcinogenicity: An Updating of IARC Monographs Volumes 1–42](https://publications.iarc.fr/139) (accessed on 20 september 2023); [Cover82.qxd (who.int)](https://monographs.iarc.who.int/wp-content/uploads/2018/06/mono82.pdf)(accessed on 14 september 2023); [IARC Publications Website - Some Naturally Occurring Substances: Food Items and Constituents, Heterocyclic Aromatic Amines and Mycotoxins](https://publications.iarc.fr/74) (accessed on 15 september 2023). |
| DON | *Fusarium lunulosporum, F. graminearum, F. culmorum, F. sporotrichioides, F. cerealis* | Grains (such as wheat and beans) | DON has been demonstrated to have severe gastrointestinal side effects, including nausea, vomiting, and diarrhoea, as well as a neurotoxic, mutagenic, and immunodepressant impact. | 1000 | 200–50 | Group 3: not carcinogenic to human | (Ahmed Adam et al., 2017; omotoya et al., 2019; Zain, 2011; ,ueno, 1984 ; [IARC Publications Website - Overall Evaluations of Carcinogenicity: An Updating of IARC Monographs Volumes 1–42](https://publications.iarc.fr/139) (accessed on 20 september 2023); [Cover82.qxd (who.int)](https://monographs.iarc.who.int/wp-content/uploads/2018/06/mono82.pdf)(accessed on 14 september 2023); [IARC Publications Website - Some Naturally Occurring Substances: Food Items and Constituents, Heterocyclic Aromatic Amines and Mycotoxins](https://publications.iarc.fr/74) (accessed on 15 september 2023). |
| Ergot alkaloids | They are poisonous alkaloid combinations secreted by *Claviceps* *species*, known pathogenic microorganisms of numerous grass *species*, notably *Claviceps purpurea and C. fusiformis.* | agricultural products like corn, wheat, rice, rye, barley, oats, and rye | ergotism, a human disease  known as St. Anthony’s fire,Effects on gastrointestinal, the central  nervous system |  |  | Not set | (Bennett & Klich, 2003). |
| Fumonisins (fumonisins B1, B2, B3, B4) | They are mycotoxins generated by *Fusarium species*, such as *Fusarium anthophilum, F. nygamai, F. proliferatum, F. dlamini, F. moniliforme, and F. verticillioides*. They have a lot in common with sphinganine, the precursor of the sphingolipid backbone; | grains (including beans, wheat, and corn), | Fumonisins have negative effects on the sphinganin/sphingosin ratio, the cell cycle, and are carcinogenic, hepatotoxic, nephrotoxic, immunosuppressive, necrotic, and immunotoxic. | 2000–4000 | 200–1000 | Group 2B: possible carcinogenic to human | (Gelineau-van Waes et al., 2005; Sun et al., 2007; Renaud et al., 2015; Rheeder et al., 2002**;** [untitled (europa.eu)](https://eur-lex.europa.eu/legal-content/EN/TXT/PDF/?uri=CELEX:02006R1881-20140701&from=EN) (accessed on 12 september 2023); [Agents Classified by the IARC Monographs, Volumes 1–134 – IARC Monographs on the Identification of Carcinogenic Hazards to Humans (who.int)](https://monographs.iarc.who.int/agents-classified-by-the-iarc/) (accessed on 20 september 2023); [IARC Publications Website - Overall Evaluations of Carcinogenicity: An Updating of IARC Monographs Volumes 1–42](https://publications.iarc.fr/139) (accessed on 20 september 2023); [Cover82.qxd (who.int)](https://monographs.iarc.who.int/wp-content/uploads/2018/06/mono82.pdf)(accessed on 14 september 2023). |
| Patulin | It is made by the fungi *P*. *expansum, Penicillium, Paecilomyces,* and *Aspergillus species* *Aspergillus clavatus, A. longivesica, A. terreus*, and *Byssochlamys sp.* | a range of fruits, vegetables, and grains, with apples, rotting maize, peanuts, figs, acha, etc. being particularly notable. | Liver, kidney, spleen, and immune system poisoning are among patulin's immediate signs. The most often reported gastrointestinal (GI) symptoms in people are nausea, vomiting, and diarrhoea. Patulin is carcinogenic, immunosuppressive, and genotoxic. | 50 | 10–50 | Group 3: not carcinogenic to human | (Rheeder et al., 2002**;** Ahmed Adam et al., 2017; Awuchi et al., 2019; [untitled (europa.eu)](https://eur-lex.europa.eu/legal-content/EN/TXT/PDF/?uri=CELEX:02006R1881-20140701&from=EN) (accessed on 12 september 2023); [Agents Classified by the IARC Monographs, Volumes 1–134 – IARC Monographs on the Identification of Carcinogenic Hazards to Humans (who.int)](https://monographs.iarc.who.int/agents-classified-by-the-iarc/) (accessed on 20 september 2023); [IARC Publications Website - Overall Evaluations of Carcinogenicity: An Updating of IARC Monographs Volumes 1–42](https://publications.iarc.fr/139) (accessed on 20 september 2023); [Cover82.qxd (who.int)](https://monographs.iarc.who.int/wp-content/uploads/2018/06/mono82.pdf)(accessed on 14 september 2023). |
| Citrinin | It has been documented in not less than 12 *Penicillium* *species* and multiple *Aspergillus* *species*, including *Penicillium verrucosum, P. citrinum, P. terreus, and P. niveus.*  *Expansum* | Wheat, barley, corn, rye, oats, and rice are examples of agricultural crops. Foods coloured with the Monascus pigment are also included. | Nephropathy, cytotoxicity, and cancer-causing properties of yellow rice illness |  |  | Group 3: not carcinogenic to human | (Bennett & Klich, 2003; Ahmed Adam et al., 2017; [untitled (europa.eu)](https://eur-lex.europa.eu/legal-content/EN/TXT/PDF/?uri=CELEX:02006R1881-20140701&from=EN) (accessed on 12 september 2023); [Agents Classified by the IARC Monographs, Volumes 1–134 – IARC Monographs on the Identification of Carcinogenic Hazards to Humans (who.int)](https://monographs.iarc.who.int/agents-classified-by-the-iarc/) (accessed on 20 september 2023); [IARC Publications Website - Overall Evaluations of Carcinogenicity: An Updating of IARC Monographs Volumes 1–42](https://publications.iarc.fr/139) (accessed on 20 september 2023) |
| Type A trichothecenes (T2 and HT2 toxin, diacetoxyscirpenol, neosolaniol) | *Cephalosporium sp., Trichoderma sp., Fusarium sporotrichioides, Fusarium graminearum, Fusarium moniliforme, Fusarium myrothecium, Fusarium acuminatum, Fusarium culmorum, and Fusarium equiseti* | Spices, coffee, and grains (including rye, oats, barley, sorghum, maize, rice, millet, and wheat) | Immunodepressants, gastrointestinal, mutagenesis activation of apoptosis in hemopoietic progenitor cells, impact on protein synthesis, and aberrant modifications to immunoglobulins are some of the side effects that might occur. |  |  | Group 3: not carcinogenic to human | (Ueno, 1984; Yuan et al., 2014; Wan et al., 2015; Adhikari et al., 2017**).** |
| Type B trichothecenes (nivalenol, deoxynivalenol, 3acetyl DON, 15acetyl DON, fusarenon X) | *Fusarium lunulosporum, F. graminearum, F. culmorum, F. sporotrichioides, F. cerealis,* and | Bananas, wheat, maize, rice, millet, sorghum, soybeans, mangoes, oats, cassava, and rye | gastrointestinal, neurotoxic, mutagenic, and immunosuppressive drugs. |  |  | Group 3: not carcinogenic to human | (Zain, 2011; ueno, 1984; Wu et al., 2020; Daud et al., 2020) |

**Table S2.** Contributing Factors to MTs Production: Effects and Required Conditions.

| **Factor** | **Effects and conditions required** | **Reference** |
| --- | --- | --- |
| Temperature and water activity | 1. Key factors for mycotoxin-producing fungi. Optimal growth and MTs production conditions vary among important MTs -producing fungal species. 2. MTs are typically produced best at 24-28°C, with some exceptions. MTs are not produced at 8°C, but *Fusarium* species produce more toxins at 12-14°C. 3. Warm conditions in tropical and subtropical areas promote AFs production, leading to MTs prevalence. 4. AFs production peaks at 33°C and activity of water 0.99. 5. MTs -producing fungi are categorized into three moisture-based groups:   - field fungi (*Alternaria and Fusarium sp.),*  -storage fungi (*Aspergillus* and *Penicillium* sp.) -Advanced decay fungi (*Fusarium*, *Trichoderma*, and *Cladosporium* *sp*.).  Field fungi thrive at 22-25% grain moisture, storage fungi prefer 13-18% (70-90% relative humidity), and advanced decay fungi grow in over 18% moisture. | Elkenany & Awad (2020);  Eugenio et al., (1970); (Bhat et al., (2010);  Fleurat-Lessard (2017); (Bankole & Adebanjo, (2003 |
| Atmosphere | The adapted atmosphere with increased carbon dioxide and decreased oxygen levels inhibits the development and MTs production of toxic fungal species. | (Elkenany & Awad, 2020) |
| Nature of substrate (foodstuffs) | 1. There are numerous foodstuffs that may be contaminated with MTs, including Tree nuts (pistachio, coconut, and walnut), oilseeds (sunflower, peanut, cottonseed, and soybean), spices (black pepper, chillies, and ginger), and cereal grains (wheat, rice, corn, maize, and sorghum), with peanuts and maize being the most vulnerable. 2. Peanuts are vulnerable to multiple fungal species during growth in soil, and the risk of fungal infection and MTs production is amplified by mechanical damage during harvest, drying, and storage. | (Elkenany & Awad, 2020) |
| Animal species | MTs, especially AFs, pose greater risks to monogastric farm animals like chickens and pigs due to their cereal-heavy diets and absence of diverse ruminal microorganisms. Ruminants, however, seem less susceptible because their rumen flora can transform MTs into less harmful or biologically inactive forms. | (Fink-Gremmels, 2008) |
| PH | 1. On an acidic pH, MTs are synthesized. 2. The pH range for both the growth and MTs production of *Aspergillus niger* is typically between 4 to 6.5. | (Sandoval-Contreras et al. (2017); Passamani et al., 2014) |
| Light | 1. UV and fluorescent light can detoxify MTs or prevent their synthesis, thereby reducing their hazardous effects.  2. MTs produced by *Fusarium verticillioides, Aspergillus parasiticus, Scopulariopsis fusca, and Verticillium lecanii* were eliminated when exposed to fluorescent light, short and long UV, and kept for three weeks at room temperature under diverse relative humidity (50-80%) | (Elkenany & Awad, 2020) |
| Other factors | MTs contamination is more prevalent in tropical and subtropical countries with inadequate infrastructure, such as transportation, storage, processing facilities, and qualified human resources. | (Elkenany & Awad, 2020) |
